# Multimodal Analysis of Aggressive Multifocal Cutaneous Squamous Cell Carcinoma Associated with a Germline COL6A3 Truncating Variant: A Case Report

**DOI:** 10.3390/diagnostics16132032

**Published:** 2026-06-29

**Authors:** Mircea Negrutiu, Stefan Cristian Vesa, Bogdan Florea, Diana Miclea, Razvan Bucur, Adrian Baican, Monica Focșan, Sorina Danescu

**Affiliations:** 1 Department of Dermatology, “Iuliu Hațieganu” University of Medicine and Pharmacy, 400012 Cluj-Napoca, Romania; negrutiu.mircea.ionut@elearn.umfcluj.ro (M.N.); adrian.baican@umfcluj.ro (A.B.); sorina.danescu@umfcluj.ro (S.D.); 2 Department of Functional Sciences, Discipline of Pharmacology, Toxicology and Clinical Pharmacology, Faculty of Medicine, “Iuliu Hațieganu” University of Medicine and Pharmacy, 400012 Cluj-Napoca, Romania; 3 Clinical Hospital of Infectious Diseases, 400000 Cluj-Napoca, Romania; bogdanflorea148@gmail.com; 4 Mother and Child Department, “Iuliu Hațieganu” University of Medicine and Pharmacy, 400012 Cluj-Napoca, Romania; diana.miclea@umfcluj.ro; 5 Medical Genetics Department, Clinical Emergency Hospital for Children, 400001 Cluj-Napoca, Romania; 6 Sanconfind Hospital, 107425 Poiana Câmpina, Romania; raz_bucur@yahoo.com; 7 Nanobiophotonics and Laser Microspectroscopy Center, Interdisciplinary Research Institute on Bio-Nano-Sciences, Babes-Bolyai University, 400271 Cluj-Napoca, Romania; monica.iosin@ubbcluj.ro

**Keywords:** cutaneous squamous cell carcinoma, high-frequency cutaneous ultrasound, fluorescence confocal microscopy, whole-exome sequencing, COL6A3 gene

## Abstract

**Background:** Cutaneous squamous cell carcinoma (cSCC) is commonly regarded as a sporadic malignancy primarily driven by ultraviolet exposure. However, the occurrence of multiple, aggressive tumors at a relatively young age suggests the presence of underlying genetic susceptibility. The role of germline variants affecting extracellular matrix organization, pigmentation pathways, and tumor metabolism in aggressive cSCC remains incompletely understood. **Case Presentation:** We describe a 53-year-old patient with a long-standing history of multiple aggressive cutaneous squamous cell carcinomas involving the scalp and facial regions, characterized by recurrent and multifocal disease. A comprehensive diagnostic approach was undertaken, including histopathological examination, fluorescence confocal microscopy, high-frequency cutaneous ultrasound, and genetic analysis using whole-exome sequencing (WES). **Results:** Histopathology confirmed high-risk features consistent with aggressive cSCC. Cutaneous ultrasound and fluorescence confocal microscopy provided complementary, non-invasive insights into tumor depth, architecture, and invasive patterns. Whole-exome sequencing identified a heterozygous truncating variant in COL6A3 (NM_004369.4:c.5645C>A, p.Ser1882Ter), classified as likely pathogenic according to ACMG criteria. Additionally, two heterozygous variants of uncertain significance were detected in TYR (NM_000372.5:c.1569C>A, p.Ser523Arg) and FH (NM_000143.4:c.1237-5_1237-4insTCTCCCTCCCTC). Although individually inconclusive, the combined germline genetic background may have contributed to the patient’s aggressive and multifocal cutaneous phenotype. **Discussion:** This case report supports a potential role of extracellular matrix remodeling, pigmentation-related susceptibility, and metabolic dysregulation in cutaneous carcinogenesis and tumor aggressiveness. This case illustrates how integrating WES with advanced non-invasive imaging techniques can enhance the understanding of biologically aggressive cSCC. **Conclusions:** This report highlights a unique case of multifocal aggressive cSCC characterized by a distinct germline genetic profile identified by WES and multimodal imaging assessment. Comprehensive molecular and imaging evaluation may be beneficial in selected patients with atypical or aggressive cutaneous squamous cell carcinoma, with implications for personalized surveillance and management.

## 1. Introduction

Cutaneous squamous cell carcinoma (cSCC), the second most common non-melanoma skin cancer (NMSC), accounts for approximately 20–25% of NMSC cases in Europe and shows a rising incidence due to population aging, cumulative ultraviolet (UV) exposure, and improved detection [1,2]. Incidence varies geographically, with higher rates reported in Western Europe compared to Central and Eastern Europe, while Romanian epidemiological data remain limited [2].

cSCC develops through a multifactorial interplay of environmental, constitutional, and genetic factors. Chronic cumulative UV radiation is the principal etiologic factor, especially in fair-skinned individuals with poor tanning ability. Male sex, advanced age, outdoor occupations, recurrent sunburns, and artificial tanning further increase risk [3]. Immunosuppression, particularly in organ transplant recipients and patients with hematologic malignancies, markedly elevates susceptibility [4]. Additional risk factors include actinic keratoses, chronic ulcers, burn scars, persistent inflammation, exposure to carcinogens such as arsenic or ionizing radiation, hereditary syndromes including xeroderma pigmentosum and epidermodysplasia verruciformis, and infection with high-risk HPV strains, especially in periungual and anogenital cSCC [5,6,7].

At the molecular level, cSCC is characterized by a high UV-induced mutational burden, predominantly involving C→T transitions. Early carcinogenic events commonly include TP53 inactivation and mutations in NOTCH1/NOTCH2, leading to impaired keratinocyte differentiation and survival of damaged cells [8,9]. Alterations in CDKN2A, RAS genes, and pathways such as PI3K/AKT/mTOR, MAPK, and TGF-β also contribute to tumor progression [9,10,11]. However, some cSCCs arise through non-UV-related mechanisms, particularly in chronic scars or inflammatory lesions, where alternative mutations such as KMT2B may predominate [12].

**Figure 1:**
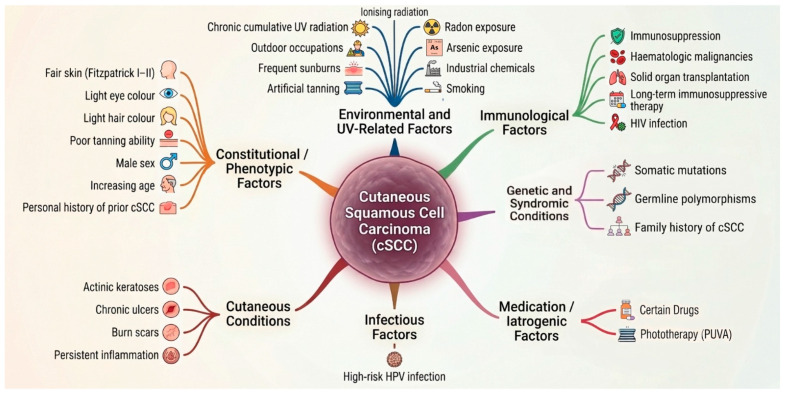
Risk factors for cutaneous Squamous Cell Carcinoma. HPV = Human Papillomavirus; UV = Ultraviolet Radiation; PUVA = Psoralen + Ultraviolet A.

The main risk factors for cSCC are illustrated in Figure 1.

Diagnosis relies primarily on clinicopathologic correlation and histopathological examination, which remains the gold standard for confirmation and risk assessment. Non-invasive imaging techniques, including dermoscopy, reflectance confocal microscopy, and optical coherence tomography, increasingly support diagnostic accuracy [13,14].

Although most primary cSCCs have an excellent prognosis when treated early, a subset of high-risk tumors may recur or metastasize, with metastasis rates of 2–5% and significantly reduced survival in advanced disease [15].

Individual cSCCs may display biological behaviors distinct from the classic UV-driven pathway due to complex environmental, molecular, and genetic interactions. Such cases highlight the genetic heterogeneity of cSCC and the role of alternative carcinogenic mechanisms. The following case emphasizes the importance of molecular analysis in identifying atypical, non-UV-related genetic alterations involved in tumor development and progression.

## 2. Case Presentation

We report the case of a 53-year-old male patient with a longstanding history of multiple aggressive cutaneous squamous cell carcinomas (cSCCs) involving the scalp and facial regions, characterized by recurrent, multifocal disease.

The patient was classified as Fitzpatrick skin phototype III. There was no history of chronic or significant occupational UV exposure. Because of a pre-existing neurological condition, the patient was largely homebound, further limiting overall sun exposure, and no additional risk factors related to prolonged outdoor activity were identified.

The family history was notable for a maternal history of multiple basal cell carcinomas (BCCs) arising after the age of 70. The patient’s past medical history included spastic paraparesis and mild intellectual disability diagnosed at 14 years of age, as well as a depressive disorder. He also had a personal history of multiple prior cSCCs and BCCs. No chronic home medications were reported.

The disease course began in 2008, at 36 years of age, with the development of a BCC in the right preauricular region, which was completely excised. In 2014, he presented with a 4.5 cm ulcerated tumor located at the vertex; histopathological examination demonstrated a moderately differentiated invasive cSCC, which was also completely excised. A preoperative non-contrast cranial computed tomography (CT) scan revealed no pathological findings. Notably, between 2014 and 2021, the patient experienced additional tumor recurrences that were surgically treated.

In early 2021, the patient presented to a plastic surgery unit with an ulcerated tumor involving the entire left auricle. Preoperative cranial and cervical CT imaging demonstrated invasion of the posterior wall of the external auditory canal. Consequently, wide local excision was performed, including resection of the left auricle and external auditory canal.

Later in 2021, the patient developed another moderately differentiated cSCC at the vertex, which was completely excised. Additionally, three further cSCCs were identified on the left nasal ala, right preauricular region, and right frontal area; all demonstrated positive surgical margins following excision. In light of these findings, the patient underwent adjuvant radiotherapy consisting of 21 sessions. Subsequently, he developed a moderately differentiated cSCC of the superior pole of the right auricle and an in situ cSCC of the nasal pyramid, both of which were completely excised.

In 2023, multiple new lesions appeared on the face and scalp. The patient reportedly received treatment with cemiplimab at a dose of 350 mg every 21 days for nine months.

In 2024, the patient presented with a cSCC involving the inferior pole of the right ear, requiring excision of the remaining auricular pavilion and external auditory canal. Attempted reconstruction with a skin graft was unsuccessful. During the same year, a basosquamous carcinoma of the left arm and a well-differentiated cSCC of the vertex were identified and completely excised. Additionally, multiple new lesions developed on the scalp and face, along with a local recurrence in the right temporoauricular region, necessitating further surgical intervention. In early 2025, the patient underwent an additional 25 sessions of radiotherapy to this region. In October 2025, systemic therapy with cemiplimab was reinitiated, with a stable course. The first course of cemiplimab, initiated in 2023, was administered for approximately nine months and subsequently discontinued. The exact reason for treatment discontinuation was not documented in the available medical records. The patient remained under clinical follow-up, and further therapeutic decisions were made based on subsequent disease evolution, which led to reinitiation of cemiplimab in 2025.

A diagnosis of multiple recurrent cutaneous squamous cell carcinomas was established; however, the markedly aggressive clinical course prompted further investigation into potential underlying factors, including a possible genetic predisposition syndrome.

**Figure 2:**
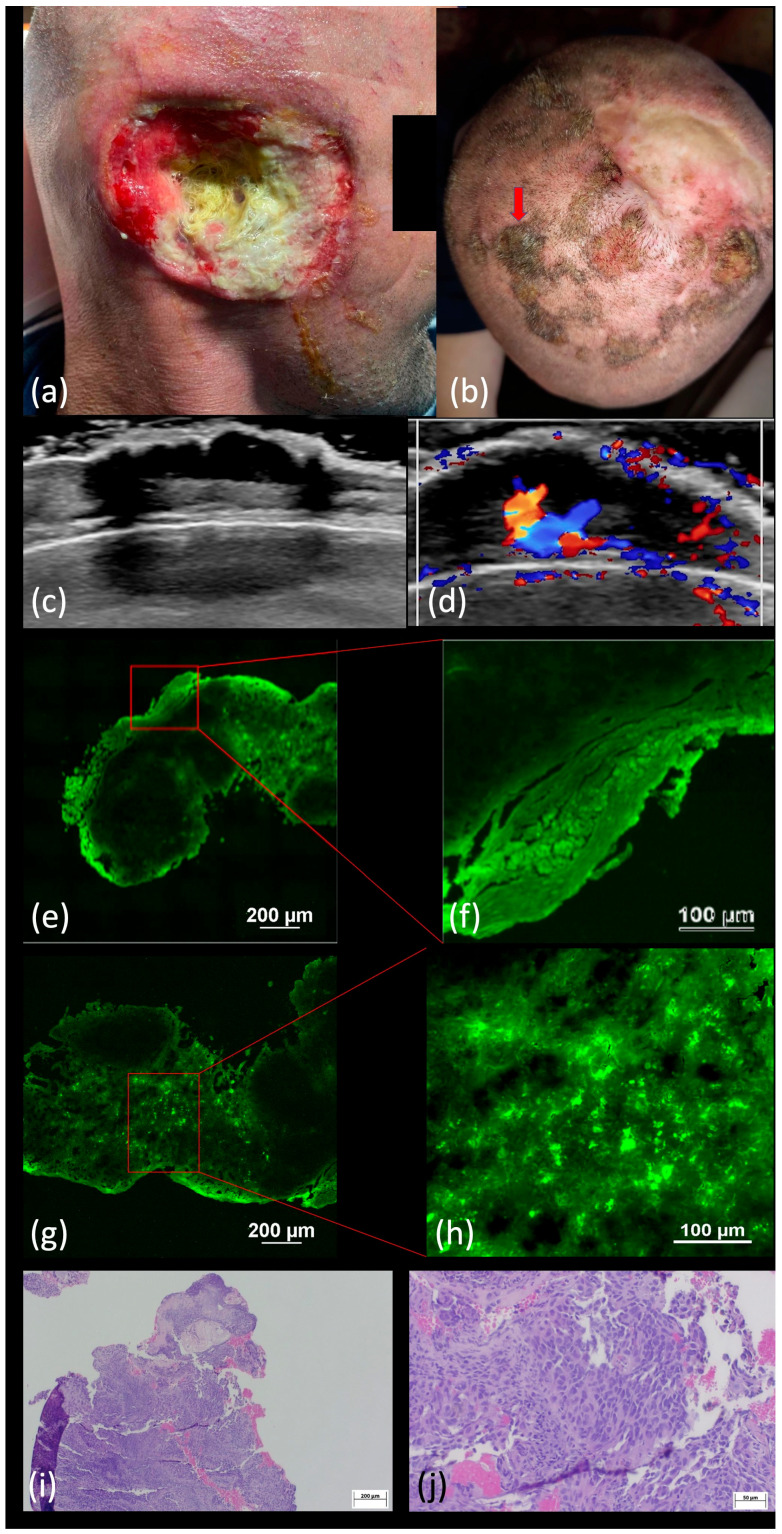
Clinical, ultrasonographic, confocal microscopic, and histopathological evaluation of a representative cutaneous squamous cell carcinoma lesion. (**a**) The clinical picture shows a right temporoauricular ulceration, covered with fibrinous deposits and exhibiting exudation, with absence of the right auricular pavilion. (**b**) Multiple ulcerated tumors covered with yellowish crusts on the scalp, with a whitish, depressed area corresponding to a prior surgical excision of a tumor. The red arrow highlights the lesion that underwent imaging evaluation and biopsy. (**c**) High-Frequency Ultrasound (20 MHz) reveals a poorly defined hypoechoic lesion with irregular contours and heterogeneous echogenicity, extending through the epidermis and dermis, associated with posterior acoustic shadowing. (**d**) Color Doppler mode shows a marked increase in local vascularization. (**e**–**h**) Ex vivo fluorescence confocal microscopy reveals a tumor characterized by hyperkeratosis and parakeratosis of the stratum corneum, accompanied by marked architectural disorganization. Numerous plump, highly reflective (bright) and speckled cells are observed within the epidermis. (**i**,**j**) The histopathological exam shows epidermis with marked hyperparakeratosis, associated with the formation of a neutrophilic abscess at this level, as well as moderate cytonuclear atypia of keratinocytes throughout the full thickness of the epidermis. The underlying dermis shows a moderately dense, diffusely distributed round-cell inflammatory infiltrate, with focal hemorrhagic extravasation.

Histopathology confirmed high-risk features consistent with aggressive cSCC. Cutaneous ultrasound and fluorescence confocal microscopy provided complementary, non-invasive insights into tumor depth, architecture, and invasive patterns. The clinical, high-frequency ultrasound (HFUS), ex vivo confocal fluorescence microscopy, and histopathological features are illustrated in Figure 2.

Whole-exome sequencing identified a heterozygous truncating variant in COL6A3 (NM_004369.4:c.5645C>A, p.Ser1882Ter), classified as likely pathogenic according to ACMG criteria. Additionally, two heterozygous variants of uncertain significance were detected in TYR (NM_000372.5:c.1569C>A, p.Ser523Arg) and FH (NM_000143.4:c.1237-5_1237-4insTCTCCCTCCCTC). Although individually inconclusive, the combined germline genetic background may have contributed to the patient’s aggressive and multifocal cutaneous phenotype.

## 3. Discussion

The literature review included in this manuscript is narrative in nature and was conducted to provide contextual background for the presented case. A non-systematic approach was used, without a predefined protocol or formal eligibility criteria. Relevant publications were identified through a targeted, non-exhaustive search of PubMed and selected peer-reviewed literature focusing on collagen VI-related disorders and cutaneous squamous cell carcinoma. Studies were selected based on their clinical and genetic relevance to the present case rather than through a formal systematic review process. Consequently, no formal evaluation of study quality or risk of bias was performed.

cSCC is a prevalent keratinocyte cancer that exhibits a wide range of clinical manifestations. A subset of cases may exhibit aggressive and recurrent behavior, particularly those occurring in the context of chronic sun exposure, immunosuppression, or genetic predisposition. However, the majority of cases have an indolent history with limited metastatic potential. Early-onset, numerous, recurrent cSCCs affecting the face and scalp make our patient’s case noteworthy and may indicate a syndromic or hereditary vulnerability [5].

A subgroup of cSCCs develop in the setting of hereditary or syndromic predispositions, sometimes presenting as early-onset, numerous, or recurring tumors, but the majority of cSCCs develop randomly. It is crucial to identify these types as they often behave aggressively and call for specialised management and monitoring techniques [15]. The principal syndromic conditions associated with multiple cSCC, including their underlying pathogenic mechanisms, modes of inheritance, clinical manifestations, and cSCC-related characteristics, are summarized in Table 1.

In addition to well-characterized genodermatoses such as xeroderma pigmentosum, epidermodysplasia verruciformis, Rothmund–Thomson syndrome, Ferguson-Smith syndrome, Muir-Torre syndrome, and oculocutaneous albinism, increasing evidence supports the existence of non-syndromic genetic susceptibility to cSCC [23,24,25]. This susceptibility is mediated by common germline variants that do not independently define a clinical syndrome but collectively contribute to cancer risk through diverse biological pathways, representing a polygenic model of disease predisposition [26].

Genome-wide association studies have identified multiple susceptibility loci, particularly in genes involved in pigmentation and UV response, including MC1R, OCA2/HERC2, SLC45A2, ASIP/RALY, IRF4, and BNC2, which influence melanin production and photoprotection and have been consistently associated with increased risk of keratinocyte cancers [23,25,27,28]. These pigmentation-related variants appear to modulate cSCC risk independently of the pigmentation phenotypes themselves, suggesting direct biological effects beyond visible phenotypic traits [29].

Additional candidate loci include genes involved in immune regulation (CADM1), anti-apoptotic pathways (AHR), keratinocyte differentiation (TP63, FOXP1), and cellular proliferation (SEC16A) [23,27]. Immune-related regions within the HLA class II locus (particularly HLA-DRB1 and HLA-DQA1) have also been identified, suggesting a role for altered tumor immune surveillance and impaired elimination of UV-damaged keratinocytes [30,31].

Collectively, these variants are better understood as low- to moderate-penetrance susceptibility factors rather than drivers of discrete hereditary syndromes, highlighting a polygenic contribution to disease risk [26,32]. Polygenic risk scores incorporating these variants have demonstrated that individuals in the highest genetic risk quintile have over threefold increased risk compared to those in the lowest quintile, with a population attributable risk of approximately 62%, suggesting that genetic predisposition accounts for a substantial proportion of cSCC cases [26,33].

More broadly, these data suggest that genetic susceptibility to cSCC may exist along a continuum, rather than as a strict dichotomy between syndromic and non-syndromic disease, and may help explain patients with multiple and aggressive cutaneous carcinomas who do not fulfill criteria for recognized syndromic entities but nonetheless demonstrate familial clustering and early-onset disease [24,32].

COL6A3 gene encodes the alpha-3 chain of type VI collagen, which is an extracellular matrix protein. This type of collagen has an important role in muscle morphologic and functional integrity. This collagen is also expressed in skin; it was demonstrated that type VI collagen also has an important role in dermal matrix structure and fibroblast function and motility, functions which are important for skin integrity, regeneration and wound healing [34]. A null mutation, as observed in our patient, may have a significant biological impact across tissues where COL6A3 is expressed; however, this remains a hypothesis that requires further validation and cannot be interpreted as evidence of a causal relationship with cutaneous squamous cell carcinoma aggressiveness. Usually, patients with COL6A3 mutations were described to have skin abnormalities, such as keratosis pilaris or abnormal scarring [35]. Type 6 collagen null variants were demonstrated to be involved in downregulation of genes with functional effects on DNA replication and repair [36].

Genetic variants of unknown significance in TYR (which code for tyrosinase enzyme, essential for melanin production in melanocytes) and FH genes (which code for fumarate hydratase—critical for the citric acid (Krebs) cycle, converting fumarate to malate to generate cellular energy) may contribute to a genetic background—via di-, tri-, or polygenic mechanisms—that worsens the clinical effects seen with harmful COL6A3 gene variants. The identified TYR and FH variants of uncertain significance should be interpreted with caution. At present, there is no functional or epidemiological evidence linking these specific variants to cutaneous squamous cell carcinoma. Therefore, their clinical relevance in the context of the present case remains unclear, and any potential contribution to disease susceptibility is purely speculative.

Although genome sequencing and functional studies could provide additional insights beyond the currently available data, these investigations are not feasible in our laboratory setting. Nevertheless, we believe that the findings derived from this case contribute valuable evidence supporting the need for further research into the role of COL6A3, including its potential involvement in severe dermatologic pathologies.

### 3.1. COL6A3 and cSCC

In the present case, the coexistence of a germline truncating COL6A3 variant together with variants of uncertain significance (VUS) in TYR and FH suggests a multi-layered susceptibility background, in which extracellular matrix (ECM) alterations, UV-related factors, and metabolic pathways may collectively contribute to tumor development. Among these, COL6A3 loss of function is hypothesized to have a central role, given the increasingly recognized contribution of tumor–stroma interactions to cSCC progression and invasiveness [37,38].

In the majority of visceral cancers studied to date (breast, bladder, colorectal, gastric, and pancreatic cancers), COL6A3 is overexpressed and acts as a pro-tumorigenic factor, promoting tumor growth, epithelial–mesenchymal transition (EMT), chemoresistance, and angiogenesis, in part through its cleaved C-terminal peptide endotrophin (ETP) [39,40,41]. In those contexts, collagen VI favors tumor progression by acting on the Akt–GSK-3β–β-catenin axis and recruiting macrophages and endothelial cells to the tumor microenvironment [40]. However, the role of collagen VI in cutaneous carcinogenesis may be fundamentally different from its role in visceral malignancies. In several visceral cancers, COL6A3 overexpression has been associated with pro-tumorigenic effects within the tumor microenvironment. In contrast, the present case involves a germline truncating variant that may result in partial loss of function. These mechanisms are biologically distinct and represent opposite molecular contexts; therefore, functional observations derived from somatic COL6A3 overexpression cannot be directly extrapolated to the germline loss-of-function setting described here. Proteomic analyses of high-risk cSCCs have identified tissue damage, ECM remodeling, and inflammation as shared microenvironmental determinants of aggressive behavior [38]. By analogy, a truncating COL6A3 variant may lead to impaired ECM organization and altered cell–matrix interactions in the skin, potentially creating a permissive microenvironment that facilitates keratinocyte invasion and tumor multiplicity [34]. It should be noted that the degree of collagen VI deficiency resulting from a heterozygous COL6A3 truncating variant may be modulated by partial compensation from the α5(VI) and α6(VI) chains, which are expressed in human skin and may form alternative heterotrimers with α1(VI) and α2(VI) in the absence of a functional α3(VI) chain [35].

This interpretation is consistent with transcriptomic data showing that null collagen VI mutations downregulate DNA replication and repair pathways, a finding particularly relevant in UV-exposed skin where efficient DNA damage repair is critical for preventing malignant transformation [36]. Additionally, collagen VI deficiency has been shown to alter ECM structure and biomechanical properties, leading to decreased tensile strength and altered collagen fibril architecture [42,43]. Recent evidence indicates that ECM stiffness and mechanical properties are directly correlated with cSCC progression, with matrix stiffening activating mechanotransduction pathways, including YAP-dependent glycolysis and Piezo1-mediated feedback loops, that promote proliferation, invasion, and EMT [44,45]. Although a COL6A3 truncating variant might be expected to decrease matrix organization rather than increase stiffness, ECM disorganization may alter mechanotransduction in complex ways, potentially creating localized microenvironmental niches with aberrant mechanical signaling [42].

Regarding the additional VUS, genome-wide association studies have confirmed TYR as a susceptibility locus for cSCC at genome-wide significance, with subsequent meta-analyses validating this association [23,27]. At the variant level, TYR Ser192Tyr (rs1042602) has been associated with SCC risk, and the TYR haplotype carrying the Arg402Gln variant allele was significantly associated with SCC risk [46]. TYR has also been shown to be a risk factor for actinic keratosis independent of skin color, suggesting pleiotropic effects combining pigmentation and oncogenic functions [47]. As for FH, germline pathogenic variants are associated with hereditary leiomyomatosis and renal cell cancer (HLRCC) syndrome, in which fumarate accumulation drives oncogenesis through pseudohypoxia and impaired DNA damage repair [48]. However, no established association between FH variants and cSCC has been reported, and the contribution of this VUS remains speculative.

The neurological phenotype observed in this patient, characterized by spastic paraparesis and mild intellectual disability, raises the possibility of a broader clinical spectrum associated with the identified germline COL6A3 truncating variant. Variants in COL6A3, encoding the α3 chain of type VI collagen, are primarily implicated in collagen VI-related myopathies, including Bethlem myopathy and Ullrich congenital muscular dystrophy. These conditions typically present with skeletal muscle involvement; however, increasing evidence suggests a wider phenotypic variability, with potential neuromuscular overlap and, in rare cases, neurodevelopmental involvement [49].

Although a formal neuromuscular or neurogenetic evaluation was not performed, the long-standing neurological symptoms documented in this patient may not be entirely incidental. The coexistence of cutaneous malignancy and a germline collagen VI pathway alteration raises the hypothesis of a possible pleiotropic effect, although a causal relationship cannot be established based on a single case. Further studies are required to clarify whether COL6A3 truncating variants may contribute to a broader multisystem phenotype beyond classical collagen VI-related myopathies.

Although the individual contribution of each variant remains uncertain, their coexistence raises the hypothesis of additive or synergistic effects that may have influenced the aggressive and multifocal clinical presentation observed in this patient. This case raises the hypothesis of a potential role for COL6A3 loss of function in cSCC susceptibility through a mechanism distinct from the pro-tumorigenic overexpression described in visceral cancers, and highlights the need for further functional studies exploring the contribution of ECM-related genes to the cutaneous tumor microenvironment [43].

### 3.2. Therapeutic Approach

#### 3.2.1. Standard of Care

First-line management of cutaneous squamous cell carcinoma relies on complete surgical excision, with Mohs micrographic surgery representing the preferred approach in high-risk tumors due to superior margin control. Radiotherapy may be used as an alternative or adjuvant modality in selected cases. However, aggressive and multifocal forms are associated with higher recurrence rates and may require systemic treatment approaches [50].

#### 3.2.2. Systemic Therapy—Immunotherapy

The treatment of advanced cSCC has undergone a dramatic change due to immune checkpoint inhibitors. The first medication authorised for locally advanced or metastatic cSCC not responsive to curative surgery or radiation therapy was the anti-PD-1 monoclonal antibody Cemiplimab, which showed clinically significant and long-lasting responses in phase II studies [51]. Reported objective response rates range around 44–47%, with durable responses in a substantial proportion of patients, highlighting its role as the current standard of care in advanced disease [52]. More recent data also support its use in neoadjuvant and adjuvant settings, with improved disease-free survival and high pathological response rates [53].

#### 3.2.3. Precision Oncology and Role of WES

The detection of germline or somatic changes that may contribute to tumor aggressiveness and treatment resistance is made possible by the use of whole-exome sequencing (WES) in clinical practice. It is becoming more well acknowledged that the pathophysiology of cSCC is multifaceted, involving genetic predisposition, changes in important regulatory mechanisms, and environmental factors such as UV exposure [54]. This genomic insight supports a shift toward precision oncology, where molecular profiling may guide risk stratification and potentially inform therapeutic decisions.

#### 3.2.4. COL6A3 and Tumor Microenvironment

The gene encodes the α3 chain of type VI collagen, a crucial structural element of the extracellular matrix (ECM); however, there are presently no targeted medicines that directly address COL6A3 changes. ECM remodelling, which promotes invasion, migration, and tumor–stroma interactions, has a significant impact on tumor development [55]. Dysregulation of ECM components may therefore contribute to a more invasive and treatment-resistant phenotype, providing a plausible biological explanation for aggressive clinical behavior in genetically predisposed cases.

##### Emerging Therapies

New treatment approaches, such as altering stromal interactions and ECM remodelling, target the tumor microenvironment. To improve medication penetration and lessen tumor invasiveness, experimental strategies including collagen-degrading nanocarriers or ECM-modifying agents are being investigated [56].

Gene-based therapeutic strategies, including CRISPR/Cas9-mediated genome editing and RNA-targeted approaches, are currently being explored in preclinical and early translational studies for inherited and acquired disorders involving extracellular matrix proteins. Recent advances in gene editing technologies have demonstrated the feasibility of correcting pathogenic variants in monogenic diseases, including those affecting collagen pathways [57,58,59].

Although these approaches have not yet been translated into clinical applications for cutaneous squamous cell carcinoma, they illustrate a potential future direction for precision medicine in genetically defined tumor subsets. At present, no clinical trials specifically target COL6A3 alterations in cSCC, highlighting an important unmet need and a promising avenue for future research.

### 3.3. Study Limitations

This study has several limitations that should be acknowledged. First, as a single case report, the findings are inherently limited in terms of external validity and cannot be generalized to broader patient populations. The interpretation of the observed clinical, genetic, and imaging features is therefore exploratory in nature.

Second, the accompanying literature review is narrative and non-systematic. It was conducted without a predefined protocol, structured search strategy, or explicit inclusion and exclusion criteria, which introduces an inherent risk of selection bias and limits reproducibility. As such, the literature synthesis should be interpreted as a qualitative and hypothesis-generating overview rather than a comprehensive systematic review.

Third, the retrospective design of the report limits the completeness of the available clinical data. Certain relevant variables, such as detailed quantitative assessment of ultraviolet exposure, occupational history, and complete documentation of therapeutic decision-making, were not fully available in the medical records.

Fourth, although whole-exome sequencing identified a germline COL6A3 truncating variant, the functional consequences of this variant in the context of cutaneous oncogenesis and the neurological phenotype remain speculative, and no functional validation studies were performed.

One additional limitation is that the exact reason for discontinuation of the first cemiplimab course administered in 2023 was not documented in the available medical records, which limits the interpretation of the overall therapeutic course and treatment response chronology.

Finally, the lack of systematic neuromuscular and neurogenetic evaluation limits the ability to establish a definitive relationship between the neurological manifestations and the identified genetic variant.

## 4. Conclusions

This report describes a unique case of multifocal, aggressive cutaneous squamous cell carcinoma associated with a germline COL6A3 truncating variant, characterized through whole-exome sequencing and multimodal imaging assessment. The coexistence of an unusual neurological phenotype and a distinct genetic alteration suggests a potential broader phenotypic spectrum related to COL6A3, although causality cannot be established based on a single case.

This case underscores the importance of integrating clinical, genetic, and imaging data in selected patients with atypical or aggressive cutaneous squamous cell carcinoma. Comprehensive molecular and imaging evaluation may contribute to improved phenotypic characterization and support more individualized diagnostic and surveillance strategies.

## Figures and Tables

**Table 1:** Genetic and syndromic conditions associated with multiple cutaneous SCCs.

Condition	Pathogenic Mechanism	Inheritance	Key Phenotype	Relevance to Present Case	Reference
*Xeroderma pigmentosum*	Defective nucleotide excision repair of UV-induced DNA damage	AR	Severe photosensitivity, early pigmentary changes, multiple early-onset cutaneous malignancies, possible neurologic involvement	Illustrates extreme UV-driven carcinogenesis due to DNA repair deficiency, highlighting contrast with cases where malignancy occurs without significant UV exposure	[16]
*Epidermodysplasia verruciformis*	Impaired immune response to HPV (EVER1/TMC6, EVER2/TMC8 mutations), UV acts as cofactor	AR	Persistent flat or verrucous HPV-related lesions resembling pityriasis versicolor	Demonstrates synergistic role of viral oncogenesis and UV exposure in SCC development	[17]
*Rothmund–Thomson syndrome*	RECQL4 helicase dysfunction leading to impaired DNA replication and genomic stability	AR	Poikiloderma, skin atrophy, skeletal abnormalities, early-onset skin malignancies	Supports association between DNA repair defects and early-onset cutaneous SCC, relevant for genetic susceptibility differential	[18]
*Ferguson–Smith syndrome*	Dysregulated TGF-β signaling (TGFBR1 pathway involvement)	AD	Multiple rapidly involuting SCC-like tumors on sun-exposed skin	Highlights paradoxical tumor regression and altered TGF-β signaling in keratinocyte tumorigenesis	[19,20]
*Muir–Torre syndrome*	DNA mismatch repair deficiency (commonly MSH2 mutation)	AD	Sebaceous tumors, keratoacanthomas, association with internal malignancies	Illustrates syndromic association between cutaneous tumors and systemic cancer predisposition	[21]
*Oculocutaneous albinism*	Defective melanin biosynthesis (TYR or TYRP1 mutations)	AR	Generalized hypopigmentation of skin, hair, eyes	Demonstrates increased susceptibility to UV-induced SCC due to lack of photoprotection	[22]

AD: Autosomal dominant; AR: autosomal recessive.

## Data Availability

The original contributions presented in the study are included in the article; further inquiries can be directed to the corresponding author.

## Abbreviations

The following abbreviations are used in this manuscript:

cSCC	cutaneous squamous cell carcinoma
NMSC	non-melanoma skin cancer
HFUS	high-frequency ultrasound
WES	whole-exome sequencing
UV	ultraviolet
Inh	inheritance
VUS	variants of uncertain significance
EMT	epithelial–mesenchymal transition
ECM	extracellular matrix
BCCs	basal cell carcinomas
